# Burning Mouth Syndrome, the Oral Microbiome, and Lactic Acid Bacteria: A Comprehensive Review of Clinical Features, Microbial Dysbiosis, and Probiotic Therapeutic Potential

**DOI:** 10.3390/microorganisms14071420

**Published:** 2026-06-29

**Authors:** Young Ju Jin, Jeong-Ah Yoon, Yoon-Jong Ryu

**Affiliations:** 1 Department of Otorhinolaryngology-Head and Neck Surgery, Kangwon National University Hospital, Kangwon National University College of Medicine, Chuncheon 24341, Republic of Korea; chindol@hanmail.net; 2 Department of Food Biotechnology and Environmental Science, Kangwon National University, Chuncheon 24341, Republic of Korea; joliejayoon@kangwon.ac.kr

**Keywords:** burning mouth syndrome, oral microbiome, lactic acid bacteria, dysbiosis, probiotics, orofacial pain, gut–brain axis

## Abstract

**Background**: Burning mouth syndrome (BMS) is a chronic orofacial pain disorder characterized by persistent intraoral burning recurring daily for at least 2 h over more than 3 months, without explanatory mucosal or laboratory findings. It affects 1.7–7.7% of the population, predominantly perimenopausal and postmenopausal women, and conventional pharmacotherapy offers only partial relief. **Aim**: This narrative review examines the associative and mechanistic evidence linking the oral microbiome to BMS and evaluates the rationale for lactic acid bacteria (LAB) as a candidate therapeutic strategy. **Methods**: PubMed/MEDLINE, Scopus, and Web of Science were searched for English-language literature on BMS, the oral microbiome, and probiotics, supplemented by mechanistic data from related conditions. **Results**: BMS patients may exhibit a compositionally distinct salivary microbiome, with reduced alpha diversity in psychiatric-comorbid subsets, though findings are heterogeneous. LAB, particularly *Lacticaseibacillus paracasei*, show antimicrobial and immunomodulatory properties relevant to oral homeostasis, but direct clinical evidence in BMS remains scarce and largely preclinical. **Conclusions**: Current evidence is predominantly cross-sectional and associative; the oral dysbiosis–BMS link and the therapeutic potential of LAB should be regarded as hypothesis-generating, warranting biomarker-anchored, strain-specific randomized trials.

## 1. Introduction

Burning mouth syndrome (BMS) is a chronic idiopathic orofacial pain disorder characterized by intraoral burning or dysesthetic discomfort persisting for more than 2 h daily for at least 3 months, in the absence of mucosal lesions or laboratory findings adequate to account for the symptoms [1,2]. Pooled epidemiological estimates indicate a general population prevalence of approximately 1.7%, rising to 7.7% among patients in clinical care settings [3]. The condition imposes a substantial functional and psychosocial burden, with a marked predilection for perimenopausal and postmenopausal women, in whom reported prevalence reaches 10–40% [4,5].

Despite decades of clinical recognition, the pathogenesis of BMS remains incompletely understood and lacks a unified mechanistic explanation. Current leading hypotheses encompass trigeminal small-fiber neuropathy with altered expression of TRPV1, NGF, P2X3, and voltage-gated sodium channels (Nav1.7, Nav1.9); central sensitization with possible nociplastic features; hormonal dysregulation involving gonadal and neuroactive steroids; micronutrient deficiencies (vitamin B12, folate, iron, and zinc); *Helicobacter pylori* infection; psychiatric comorbidities including anxiety and depression; and—most relevant to the present review—oral microbial dysbiosis [6,7,8,9]. Conventional pharmacological strategies, including topical and systemic clonazepam, alpha-lipoic acid, gabapentinoids, tricyclic antidepressants, and capsaicin, yield only partial and inconsistent symptom relief, and their adverse-effect profiles constrain long-term tolerability [10,11]. The lack of disease-modifying treatments has prompted renewed investigation into biological substrates that are both upstream of symptom generation and amenable to therapeutic intervention—foremost among which is the oral microbiome.

The human oral cavity supports the second largest microbial community in the body, encompassing more than 700 bacterial species organized into spatially structured biofilms across distinct ecological niches, including saliva, tongue dorsum, supra- and subgingival plaque, buccal mucosa, hard and soft palate, and tonsils [12,13]. Under healthy conditions, the oral microbiome is dominated by genera such as *Streptococcus*, *Veillonella*, *Neisseria*, *Actinomyces*, *Rothia*, *Haemophilus*, and *Prevotella*, with site-specific tropism giving rise to stable yet compositionally distinct microenvironments [13]. Disruption of this homeostatic balance—referred to as oral dysbiosis—has been associated with caries, periodontitis, oral cancer, mucosal disorders, and, increasingly, chronic orofacial pain conditions, including BMS [7,14,15]. Although the oral microbiome literature specific to BMS remains limited, an emerging consensus suggests that BMS patients exhibit reproducible compositional shifts and, in subsets stratified by pain intensity or psychiatric comorbidity, reduced alpha diversity [16,17].

Lactic acid bacteria (LAB)—encompassing the genera *Lactobacillus*, *Bifidobacterium*, *Streptococcus*, and *Lactococcus*—are Gram-positive, fermentative microorganisms that produce lactic acid as the principal end-product of carbohydrate catabolism [18,19]. Certain species are indigenous to the human oral cavity, whereas others are introduced through dietary sources such as fermented dairy products, vegetables, and soybean-based foods, or administered as exogenous probiotic supplements [14,20]. Classified as Generally Recognized as Safe (GRAS), LAB have an established record of use in both food production and clinical settings [18,21]. Among LAB, *Lacticaseibacillus paracasei* (formerly *Lactobacillus paracasei*) [22] has emerged as a particularly promising candidate for oral health applications, with whole-genome sequencing of multiple strains revealing conserved core functions in carbohydrate metabolism, cellular adhesion, and stress tolerance [23,24].

The present review integrates current evidence across three rapidly advancing fields: (i) the clinical features and etiopathogenesis of BMS, with emphasis on neuropathic, hormonal, nutritional, and microbial mechanisms; (ii) the composition, dynamics, and dysbiotic alterations of the oral microbiome in BMS; and (iii) the mechanistic and clinical evidence supporting LAB—particularly *L. paracasei*—as potential modulators of oral mucosal homeostasis and orofacial pain. Special attention is directed toward translational links among yeast-derived metabolites, short-chain fatty acids, GABAergic signaling, and trigeminal nociceptor function, which together constitute a biologically plausible oral-gut–brain axis through which probiotic interventions may exert antinociceptive effects. We conclude by identifying critical knowledge gaps—including the predominance of cross-sectional associative data and the paucity of BMS-specific probiotic RCTs—and propose a methodological framework and research agenda for biomarker-anchored, strain-specific clinical trials designed to move the field from association toward mechanism.

### Search Strategy

This article is a narrative review. To enhance transparency and minimize the risk of selection bias, the literature underpinning this review was identified through a structured, non-systematic search of the PubMed/MEDLINE, Scopus, and Web of Science databases for English-language articles published from database inception to May 2026. The search combined Medical Subject Headings (MeSH) and free-text terms relating to three thematic domains, using Boolean operators: (i) the clinical entity—“burning mouth syndrome”, “stomatodynia”, “glossodynia”, “oral dysesthesia”; (ii) the microbial domain—“oral microbiome”, “oral microbiota”, “dysbiosis”, “salivary microbiome”, “*Candida*”; and (iii) the therapeutic domain—“lactic acid bacteria”, “probiotics”, “*Lactobacillus*”, “*Lacticaseibacillus paracasei*”, “postbiotics”. Reference lists of relevant articles and reviews were screened manually to identify additional sources (snowballing). Priority was given to peer-reviewed original research, systematic reviews, and meta-analyses; given the limited number of studies addressing the oral microbiome and probiotic intervention specifically in BMS, mechanistic and clinical evidence derived from in vitro studies, animal models, and related oral and systemic conditions was also incorporated and is explicitly identified as such throughout the text. Articles were selected on the basis of relevance to the review’s thematic scope, methodological soundness, and contribution to the mechanistic narrative, with preference for the most recent and authoritative sources where overlapping evidence existed. Non-English publications, conference abstracts without full text, and non-peer-reviewed sources were excluded.

## 2. Burning Mouth Syndrome: Clinical Features and Etiopathogenesis

### 2.1. Definition, Classification, and Diagnostic Criteria

Under the third edition of the International Classification of Headache Disorders (ICHD-3), BMS is operationally defined as “an intraoral burning or dysaesthetic sensation, recurring daily for more than 2 h per day for more than 3 months, without clinically evident causative lesions” [1]. Complementary nosological frameworks—the International Classification of Orofacial Pain [2] and the ICD-11 criteria endorsed by the International Association for the Study of Pain (IASP)—introduce a clinically important dichotomy between primary BMS, classified as a chronic primary pain disorder, and secondary BMS arising from identifiable local or systemic pathology such as oral candidiasis, lichen planus, hyposalivation, haematinic deficiencies, diabetes mellitus, Sjögren syndrome, or causative medications [7,8]. The IASP scheme also allows stratification based on whether quantitative sensory testing (QST) reveals somatosensory abnormalities, thereby capturing heterogeneity in underlying neuropathic phenotypes [2]. To reduce ongoing diagnostic ambiguity, an international Delphi process has proposed revisions to BMS terminology and ICD-11 diagnostic thresholds [25].

Diagnosis is principally one of exclusion: clinically evident mucosal lesions, systemic conditions, and medications known to cause oral burning must be ruled out through history, intraoral examination, salivary flow assessment, complete blood count, fasting glucose or HbA1c, vitamin B12, folate, ferritin, zinc, autoantibody screening when indicated, and *Candida* culture or smear [11,26].

### 2.2. Epidemiology

A large-scale systematic review and meta-analysis of 41 studies comprising over 26,000 participants estimated the global pooled prevalence of BMS at 1.73% in the general population and 7.72% among patients in clinical care settings, with substantial regional heterogeneity: Asia 1.05%, Europe 5.58%, and North America 1.10% [3]. A subsequent meta-analysis conducted under ICHD diagnostic criteria documented a marked female predominance, with female-to-male ratios ranging from approximately 3:1 to 7:1, peak incidence concentrated in the sixth and seventh decades of life, and statistically significant associations with comorbid anxiety, depression, and suboptimal oral hygiene [8]. Emerging evidence suggests that the COVID-19 pandemic may have contributed to an increase in BMS-like presentations: the frequency of COVID-19-associated oral burning has been estimated at approximately 4% in mild-to-moderate cases and 15% in severely hospitalized patients [27], while a survey of individuals with long-COVID found that 78% reported at least one oral manifestation, including burning mouth sensation and dysgeusia [28].

### 2.3. Clinical Presentation

The hallmark presentation of BMS is a persistent or near-persistent sensation of burning, scalding, or stinging within the oral cavity, with the anterior two-thirds of the tongue—particularly the tongue tip and lateral borders—representing the most frequently affected site, followed in descending order by the hard palate, lips, and buccal mucosa. A characteristic diurnal pattern is observed: symptom intensity tends to be relatively mild upon waking and escalates progressively throughout the day, with transient relief commonly reported during eating, drinking, or chewing—a feature that helps distinguish BMS from other chronic orofacial pain conditions [26]. A substantial proportion of patients—estimated at 70–80%—concurrently report dysgeusia, manifesting as persistent metallic, bitter, or otherwise altered taste perception, and xerostomia, defined as the subjective sensation of oral dryness; notably, objective measurement of salivary output reveals true hyposalivation in only a minority of affected individuals [29,30]. Beyond the primary oral symptoms, sleep disturbance, mood disorders, and measurable reductions in oral health-related quality of life frequently co-occur and contribute substantially to the overall disease burden [8].

### 2.4. Pathophysiological Mechanisms

**Figure 1:**
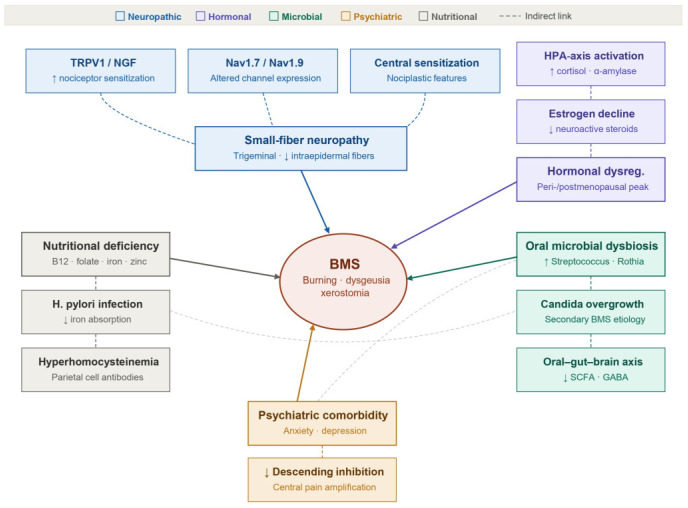
BMS: multifactorial pathophysiology.

The pathophysiology of BMS is multifactorial and incompletely understood, with several interrelated mechanisms proposed to contribute to symptom generation and maintenance. These encompass peripheral and central neuro-pathic alterations, hormonal and neuroactive steroid dysregulation, nutritional and endocrine deficiencies together with infectious contributors, psychiatric comorbidity, and disturbances of salivary function and composition. Ra-ther than acting in isolation, these mechanisms are increasingly recognized to interact within a convergent network that culminates in the characteristic burning sensation, providing the conceptual framework within which the po-tential role of the oral microbiome—the principal focus of this review—can be situated. An integrated overview of these contributing mechanisms is presented in Figure 1, and each is examined in turn in the subsections below.

#### 2.4.1. Peripheral and Central Neuropathic Mechanisms

The neuropathic basis of BMS was first substantiated histopathologically by Lauria et al. [6], who reported a marked reduction in epithelial and subpapillary nerve fiber densities in tongue biopsies from BMS patients compared with matched controls, providing the first direct evidence of trigeminal small-fiber neuropathy in this disorder. These foundational observations were subsequently corroborated and expanded by a comprehensive systematic review, which cataloged a range of neurobiological abnormalities, including immunohistochemical upregulation of fibers expressing TRPV1, NGF, and P2X3; overexpression of the voltage-gated sodium channel Nav1.7 accompanied by modest downregulation of Nav1.9 mRNA; prolonged blink-reflex latencies; and corneal confocal microscopy findings indicative of peripheral nerve fiber loss [7]. Complementary evidence from quantitative sensory testing (QST) reveals elevated cool-detection thresholds and increased cold-pain thresholds in BMS patients, a somatosensory profile that closely parallels that of established small-fiber neuropathy conditions, including diabetic peripheral neuropathy and fibromyalgia. In parallel with the peripheral neuropathic framework, a nociplastic pain model has been proposed to account for central pain-processing dysregulation observed in a clinically meaningful subset of BMS patients, suggesting that aberrant central sensitization may contribute independently to symptom generation and maintenance [31].

#### 2.4.2. Hormonal and Neuroactive Steroid Dysregulation

The marked female preponderance and the clustering of BMS onset around the peri- and postmenopausal transition implicate gonadal steroid-dependent modulation of oral nociceptive processing as a key pathophysiological contributor. Estrogen receptors are widely expressed across oral mucosal epithelium, salivary gland tissue, and trigeminal sensory neurons, and the precipitous decline in circulating estrogen accompanying menopause is associated with dysregulated synthesis of neuroactive steroids at both mucosal and central nervous system levels, potentially lowering the excitability threshold of trigeminal and glossopharyngeal primary afferents [4,5]. This hormonal vulnerability is further compounded by the effects of chronic psychological stress and anxiety, which dysregulate hypothalamic–pituitary–adrenal (HPA) axis activity and alter adrenal glucocorticoid output, thereby amplifying the underlying neuroendocrine imbalance and potentially perpetuating central sensitization.

#### 2.4.3. Nutritional, Endocrine, and Infectious Contributors

Deficiencies in hematinic micronutrients—specifically vitamin B12, folate, and iron—as well as zinc insufficiency, are disproportionately prevalent in BMS patient cohorts, and symptom improvement following nutritional repletion has been reported in a proportion of affected individuals, lending support to their designation as reversible causes of secondary BMS [9,32]. In subsets of patients presenting with iron deficiency, concomitant hyperhomocysteinemia and serological evidence of gastric parietal cell autoimmunity have also been documented, suggesting an underlying autoimmune or malabsorptive mechanism [32]. An elevated prevalence of *Helicobacter pylori* infection among BMS patients has led to the hypothesis that this pathogen may indirectly contribute to oral mucosal dysesthesia. The proposed mechanism centers on *H. pylori*-induced chronic atrophic gastritis, which progressively impairs gastric acid secretion and thereby compromises the luminal absorption of iron and vitamin B12—micronutrients whose deficiency is independently implicated in BMS pathogenesis [32,33]. A secondary pathway involves ectopic oral colonization by *H. pylori*, which may sustain a chronic, low-grade proinflammatory cytokine environment, potentially sensitizing trigeminal nociceptors at the mucosal level. However, a definitive causal relationship between *H. pylori* infection and BMS symptomatology has yet to be established, and prospective eradication trials with rigorous symptom endpoints are needed to resolve this question.

#### 2.4.4. Psychiatric Comorbidity

Comorbid anxiety and depression occur at elevated rates among BMS patients, a pattern corroborated by meta-analytic evidence demonstrating statistically robust associations between BMS and both mood disorders [8]. The precise nature of this relationship remains a subject of ongoing debate: psychological distress may precede and contribute to the development of BMS through neuroendocrine and central sensitization mechanisms, may arise as a consequence of chronic pain and functional impairment, or may interact bidirectionally in a self-perpetuating cycle that amplifies both symptom burden and affective dysregulation [8]. Regardless of directionality, the extensively characterized reciprocal relationship between mood state and nociceptive processing—mediated through descending corticolimbic modulatory pathways, hypothalamic–pituitary–adrenal axis dysregulation, and neuroactive metabolites of gut–brain axis origin—provides a compelling mechanistic rationale for treatment approaches that integrate psychological intervention with pharmacological strategies targeting both pain and affective comorbidity [8,31].

#### 2.4.5. Salivary Function and Composition

Despite the absence of clinically overt xerostomia in the majority of BMS patients, converging evidence from multiple independent studies indicates a modest but measurable reduction in unstimulated salivary flow rates in affected cohorts relative to healthy controls (mean 0.11 mL/min versus 0.21 mL/min), with approximately 46% of patients fulfilling established criteria for hyposalivation and a substantial proportion concurrently experiencing subjective oral dryness [29,30,34]. At the biochemical level, salivary concentrations of cortisol and α-amylase are consistently elevated in BMS, a pattern interpreted as a reflection of heightened sympathoadrenal and hypothalamic–pituitary–adrenal axis activation in response to chronic pain and psychological stress; by contrast, salivary cytokine profiles—including IL-2, IL-6, IL-18, and TNF-α—have yielded less reproducible findings across studies, likely attributable to methodological heterogeneity and patient selection differences [35,36]. Emerging evidence from salivary metabolomics has begun to delineate a molecular signature characteristic of BMS, with downregulation of caffeine metabolic pathways and reduced concentrations of tyrosine pathway intermediates—including L-dopa, L-tyrosine, and tyramine—identified as potentially discriminating biochemical features that distinguish BMS patients from unaffected controls [37].

### 2.5. Current Management

Current BMS management is inherently multimodal and requires individualization according to the patient’s clinical phenotype, comorbidity profile, and predominant pathophysiological mechanism. A comprehensive systematic review of available therapeutic options concluded that topical clonazepam, alpha-lipoic acid at doses of 300–800 mg/day, low-level laser therapy, capsaicin administered topically or as an oral rinse, and cognitive-behavioral therapy (CBT) demonstrate the most consistent evidence for both short- and long-term symptom attenuation, and are generally associated with acceptable tolerability and mild adverse-effect profiles [38]. Combination regimens incorporating clonazepam alongside alpha-lipoic acid have been associated with additive symptomatic benefit, while CBT has demonstrated particularly durable efficacy in patients with prominent comorbid anxiety or depressive disorders, likely through its capacity to modulate descending pain inhibitory pathways and maladaptive pain-related cognitions [26,39]. Nevertheless, no currently available monotherapy or combination regimen reliably achieves complete and sustained remission across the heterogeneous BMS population, underscoring an unmet therapeutic need and providing a strong rationale for investigating biologically targeted interventions—including microbiome-modulating strategies designed to correct underlying oral mucosal and systemic dysbiosis—as adjunctive or alternative treatment approaches. Critically, the limited efficacy of current empirical regimens reflects not only the absence of disease-modifying agents but also a deeper conceptual limitation: the treatment of a mechanistically heterogeneous disorder as though it were a homogeneous syndrome [7,26,38] (Table 1).

### 2.6. Evolution of BMS Management Paradigms

**Figure 2:**
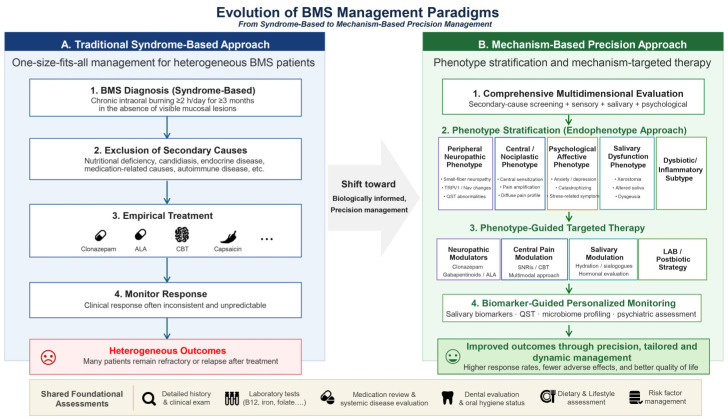
Evolution of BMS management paradigms: from syndrome-based to mechanism-based precision management. (**A**) The traditional syndrome-based approach applies a uniform diagnostic and empirical treat-ment sequence to all patients, producing heterogeneous outcomes. (**B**) The emerging mecha-nism-based precision approach stratifies patients into distinct endophenotypes (peripheral neu-ropathic, central/nociplastic, psychological/affective, salivary dysfunction, and dysbi-otic/inflammatory) to guide targeted therapy and biomarker-anchored monitoring.

The foregoing limitations of syndrome-based, exclusion-driven management have catalyzed a conceptual shift toward mechanism-based precision medicine in BMS (Figure 2). The traditional paradigm (Figure 2A) applies a uniform diagnostic and therapeutic sequence—secondary-cause exclusion followed by empirical pharmacotherapy with clonazepam, alpha-lipoic acid (ALA), cognitive-behavioral therapy (CBT), or capsaicin—irrespective of the patient’s underlying pathophysiology. This one-size-fits-all approach produces heterogeneous and frequently unsatisfactory outcomes, with a substantial proportion of patients remaining refractory or relapsing after initial treatment [26,38].

The emerging mechanism-based precision approach (Figure 2B) proposes replacing uniform empirical treatment with a structured multidimensional evaluation that integrates quantitative sensory testing (QST), salivary function assessment, microbiome profiling, and psychological screening alongside standard secondary-cause exclusion [7,26,38]. On this basis, patients are stratified into distinct endophenotypes: a Peripheral Neuropathic Phenotype characterized by small-fiber neuropathy, TRPV1/Nav channel alterations, and QST abnormalities [6,7]; a Central/Nociplastic Phenotype defined by central sensitization, pain amplification, and diffuse pain profiles [31]; a Psychological/Affective Phenotype with prominent anxiety, depression, catastrophizing, and stress-related symptoms [8,17]; a Salivary Dysfunction Phenotype presenting with xerostomia, altered salivary composition, and dysgeusia [30,34]; and a Dysbiotic/Inflammatory Subtype associated with compositional shifts in the oral microbiome and heightened mucosal inflammatory tone [16,17]. Each endophenotype informs targeted therapeutic selection: neuropathic modulators (clonazepam, gabapentinoids, ALA) for peripheral phenotypes; SNRIs and multimodal CBT for central and psychological phenotypes; sialogogues and hormonal evaluation for salivary phenotypes; and lactic acid bacteria (LAB)/postbiotic strategies for dysbiotic subtypes [38,44,46]. This phenotype-guided approach is further complemented by biomarker-anchored monitoring—incorporating salivary cortisol, inflammatory cytokines, QST, microbiome profiling, and psychiatric assessment—enabling dynamic and responsive management throughout the clinical course [35,36,47]. Shared foundational assessments, including detailed clinical history, targeted laboratory screening (vitamin B12, iron, folate), medication review, dental evaluation, and dietary assessment, remain integral to both paradigms but serve in the precision model as the springboard for individualized stratification rather than as therapeutic endpoints in themselves [9,32]. This evolution from phenotype-agnostic exclusion to mechanism-anchored endophenotyping represents the direction toward which future BMS clinical trial design, biomarker development, and therapeutic innovation—including the oral microbiome-targeting strategies reviewed in subsequent sections—should be systematically oriented [7,25,38].

## 3. The Oral Microbiome in Health and BMS

### 3.1. Composition and Site-Specific Architecture of the Healthy Oral Microbiome

Cataloged within the Human Oral Microbiome Database, the healthy oral microbiome encompasses more than 700 phylotypes distributed across five major phyla: Firmicutes (*Streptococcus*, *Veillonella*, *Granulicatella*), Proteobacteria (*Neisseria*, *Haemophilus*), Actinobacteria (*Rothia*, *Corynebacterium*, *Actinomyces*), Bacteroidetes (*Prevotella*, *Porphyromonas*, *Capnocytophaga*), and Fusobacteria (*Fusobacterium*) [12]. Each oral anatomical niche—saliva, tongue dorsum, buccal mucosa, hard palate, and supra- and subgingival plaque—sustains a reproducibly distinct microbial community shaped by local physicochemical gradients and nutrient availability. The tongue dorsum warrants particular emphasis in the BMS context: high-resolution spatial imaging has demonstrated that its microbial architecture consists of structured multispecies consortia anchored to epithelial cell surfaces, with tongue-tropic *Streptococcus* species (*S. salivarius*, *S. parasanguinis*, *S. infantis*, *S. australis*) occupying central positions within these biogeographic arrays [13]. Because saliva continuously samples shed cells and planktonic bacteria from all niche communities, it serves as a practical and representative specimen for clinical microbiome investigations [14].

Defining a universally shared “core” oral microbiome has proven challenging because of inter-individual variability, but a panel of genera—*Streptococcus*, *Veillonella*, *Granulicatella*, *Neisseria*, *Haemophilus*, *Rothia*, *Fusobacterium*, *Prevotella*, and *Porphyromonas*—is consistently identified across healthy adults, with aggregate *Streptococcus* abundances of 12–23% across saliva, tongue, plaque, and oral rinse [14,48].

### 3.2. Microbiome Dysbiosis in Burning Mouth Syndrome

**Figure 3:**
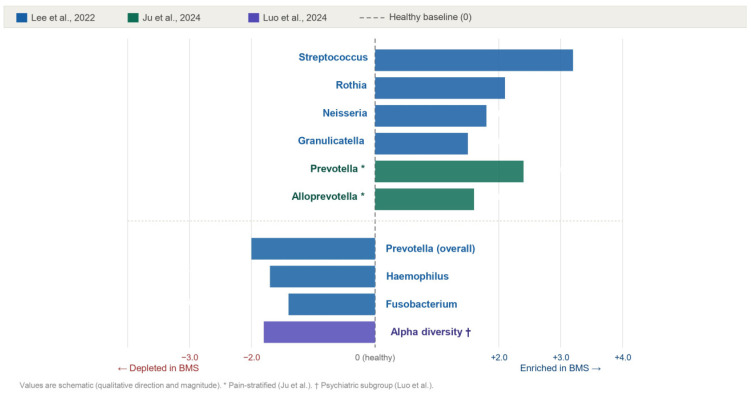
Comparison of oral microbiome composition in healthy individuals and BMS patients [16,17,47].

The intersection of BMS and oral microbiome research constitutes a rapidly developing area of inquiry, and accumulating evidence from independent 16S rRNA amplicon sequencing and shotgun metagenomic studies has begun to delineate a recognizable pattern of salivary dysbiosis in affected individuals. In a case–control investigation employing V3–V4 region amplicon sequencing of unstimulated whole saliva obtained from 19 primary BMS patients and 22 healthy volunteers, Lee et al. [16] documented relative enrichment of *Streptococcus*, *Rothia*, *Neisseria*, and *Granulicatella* in the BMS salivary microbiome, with concurrent depletion of *Prevotella*, *Haemophilus*, and *Fusobacterium*. In a study specifically examining the psychiatric-comorbid BMS subpopulation, Luo et al. [17] identified significantly reduced alpha diversity, as quantified by Shannon and Chao1 indices, accompanied by salivary metabolomic profiles indicative of perturbation in amino acid and nucleotide biosynthetic pathways. Further stratification by pain severity revealed that individuals classified as high-pain BMS harbored higher relative abundances of Prevotella and Alloprevotella than both low-pain BMS patients and healthy controls, suggesting that salivary microbial community composition may track clinical symptom intensity [47]. Importantly, reductions in alpha diversity are not a universal finding across BMS populations; rather, they appear most pronounced in subgroups characterized by concurrent psychiatric comorbidity or elevated pain burden, whereas aggregate case–control analyses yield heterogeneous and sometimes conflicting results [7]. The individual oral microbiome studies in BMS discussed above are summarized in Table 2, and the taxonomic and alpha-diversity shifts identified across these studies are synthesized and compared by genus and study source in Figure 3, which highlights both cross-study recurring patterns and subgroup-specific variations in microbial composition.

While a consensus dysbiotic signature remains to be established, the cumulative evidence consistently supports three overarching themes: (i) relative enrichment of *Streptococcus* is the most reproducible compositional feature of the BMS salivary microbiome across independent cohorts; (ii) microbial compositional shifts exhibit meaningful correlations with both pain intensity and psychiatric comorbidity burden; and (iii) accompanying functional metabolomic disturbances raise the possibility that oral dysbiosis may be associated with altered local concentrations of metabolites capable of modulating trigeminal nociceptor activity [17,37].

It must be emphasized, however, that essentially all available microbiome data in BMS derive from cross-sectional, case–control studies with modest sample sizes. Such designs can establish association but cannot determine the direction of causality, and they do not permit the conclusion that dysbiosis is a cause—rather than a consequence or epiphenomenon—of BMS. Observed microbial differences may equally reflect secondary effects of reduced salivary flow, altered oral pH, medication use, dietary modification, or psychiatric comorbidity that frequently accompany the condition. Accordingly, the associations described above should be interpreted as suspected relationships requiring confirmation through prospective, longitudinal, and interventional studies rather than as evidence of a definitive causal role.

### 3.3. Candida Species, Oral Candidiasis, and BMS

*Candida albicans* is the most prevalent fungal commensal in the human oral cavity, with colonization rates ranging from 20% to 60% among immunocompetent adults. Symptomatic oral candidiasis represents a well-established cause of secondary BMS and is of particular diagnostic significance because it can produce a burning sensation clinically indistinguishable from that of primary BMS in the absence of visible mucosal lesions or classical pseudomembranous plaques, thereby necessitating microbiological confirmation via *Candida* culture or cytological smear as a mandatory component of the routine BMS diagnostic workup [49].

The growing prevalence of multidrug-resistant *Candida auris*, including its documented capacity for oral mucosal colonization, has substantially intensified interest in non-antifungal therapeutic strategies—among which LAB-based probiotic interventions have emerged as particularly promising candidates—for both the prophylaxis and adjunctive clinical management of oral candidiasis [50,51].

### 3.4. Salivary Biomarkers, Metabolomics, and Functional Outputs

Extending beyond taxonomic characterization, functional interrogation of the oral microenvironment in BMS yields complementary and mechanistically informative insights. Salivary concentrations of cortisol and α-amylase are reproducibly elevated in BMS patients relative to healthy controls, a biochemical pattern consistent with chronic upregulation of hypothalamic–pituitary–adrenal (HPA) axis activity and heightened sympathoadrenal tone; by contrast, salivary cytokine profiles—encompassing IL-2, IL-6, IL-18, and TNF-α—exhibit considerable inter-study variability, likely reflecting heterogeneity in patient phenotype, sampling protocols, and analytical methodology [35,36]. Untargeted salivary metabolomics has further revealed suppression of caffeine catabolic pathways and dysregulation of tyrosine pathway intermediates in BMS patients compared with controls, pointing toward broader disruptions in aromatic amino acid metabolism that may have downstream consequences for neurotransmitter biosynthesis and mucosal nociceptive signaling [37]. Collectively, these functional biochemical readouts delineate a salivary phenotypic substrate that is both biologically plausible as a target for microbiome-modulating interventions and sufficiently well-characterized to serve as a reference framework against which the effects of LAB-based probiotic administration can be systematically benchmarked in future mechanistic and clinical trials.

## 4. Lactic Acid Bacteria as Therapeutic Modulators of the Oral Cavity

### 4.1. General Properties and Taxonomy of LAB Relevant to Oral Health

The term “lactic acid bacteria” refers to a phenotypically heterogeneous and phylogenetically broad grouping of Gram-positive, non-spore-forming, facultatively anaerobic microorganisms that share the defining metabolic characteristic of producing lactic acid as the principal end-product of carbohydrate fermentation. Within the context of oral health, the genera of greatest clinical relevance include the extensively reclassified *Lactobacillus* sensu lato—a taxon that has undergone major taxonomic reorganization and now encompasses approximately 25 distinct genera, among them *Lacticaseibacillus* and *Limosilactobacillus*—alongside *Bifidobacterium*, *Streptococcus* (notably *S. salivarius*, which occupies a prominent ecological role on the tongue dorsum), *Lactococcus*, and *Weissella*. Within the oral ecosystem, certain LAB taxa function as autochthonous residents, whereas others are introduced transiently through consumption of fermented dietary products—including yogurt, kefir, kimchi, and doenjang—or via deliberately formulated exogenous probiotic preparations. The vast majority of LAB species used in clinical and experimental research have been granted Generally Recognized as Safe (GRAS) status by the United States Food and Drug Administration, a designation that reflects their extensive history of safe human consumption across diverse cultural and dietary contexts [18,19].

### 4.2. Antimicrobial Mechanisms

**Figure 4:**
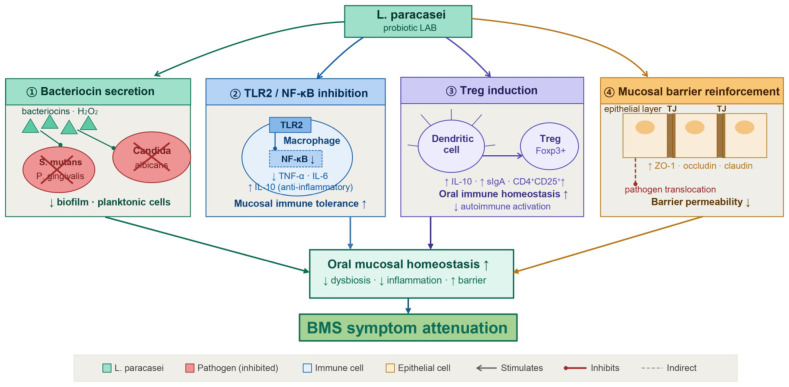
Antimicrobial and immunomodulatory mechanisms of *Lacticaseibacillus paracasei*.

LAB exert antagonistic activity against oral pathogens through several mechanistically distinct and complementary pathways: (i) elaboration of short-chain organic acids—including lactic, acetic, and propionic acid—which reduce local environmental pH and selectively inhibit acid-sensitive pathogenic species; (ii) generation of reactive oxygen species, principally hydrogen peroxide, with broad-spectrum antimicrobial activity; (iii) biosynthesis and secretion of proteinaceous bacteriocins and bacteriocin-like inhibitory substances; (iv) competitive exclusion of pathogens from mucosal and dental surfaces through occupation of adhesion receptors and interference with biofilm establishment; and (v) physical co-aggregation with target pathogens, facilitating their mechanical clearance from the oral cavity [52,53]. Among LAB, *Lacticaseibacillus paracasei* strains have been shown to elaborate bacteriocins spanning a molecular weight range of 3–56 kDa, with documented inhibitory activity against *Porphyromonas gingivalis*, *Streptococcus mutans*, and additional clinically relevant oral pathogens [50]. Notably, in vitro studies have shown that postbiotic preparations derived from *L. paracasei* 28.4 have demonstrated the capacity to inhibit *Candida albicans* in both planktonic and sessile biofilm states, including recalcitrant persister cell populations, and additionally exhibit antifungal activity against the emergent multidrug-resistant pathogen *Candida auris* [51]. Cell-free culture supernatants of *Ligilactobacillus salivarius* have been shown to attenuate *S. mutans* biofilm formation by downregulating phosphoenolpyruvate-dependent phosphotransferase systems, ATP-binding cassette (ABC) transporters, two-component regulatory systems, and exopolysaccharide biosynthetic gene clusters; bioactive compounds responsible for these effects include phenyllactic acid, sorbitol, and honokiol [54]. *Limosilactobacillus fermentum* strains of human oral origin have similarly been reported to suppress *S. mutans* proliferation and virulence through the combined action of reuterin and organic acid production, further expanding the breadth of the antimicrobial repertoire available within the LAB clade [55]. The multifaceted therapeutic potential of *L. paracasei* at the oral mucosal interface—encompassing direct pathogen suppression, antifungal activity, and downstream modulation of host mucosal immune responses—is synthesized and schematically illustrated in Figure 4.

### 4.3. Immunomodulatory Effects

LAB modulate both innate and adaptive immune circuitry through engagement of pattern-recognition receptors—including Toll-like receptor 2 (TLR2), TLR9, and nucleotide-binding oligomerization domain (NOD) receptors—thereby driving context-dependent cytokine reprogramming, facilitating the expansion of immunoregulatory T-cell subsets, and enhancing secretory immunoglobulin A (sIgA) production at mucosal surfaces. At the strain-specific level, selected *L. paracasei* strains have been shown to attenuate lipopolysaccharide-induced secretion of TNF-α and IL-6 from human peripheral blood mononuclear cells through upregulation of endogenous negative regulators of the NF-κB signaling cascade via a TLR2–IRAK4-dependent mechanism, thereby dampening proinflammatory cytokine output without global immunosuppression [56]. In alignment with this anti-inflammatory phenotype, *L. paracasei* MSMC39-1 has been reported to reduce TNF-α production in intestinal epithelial cell models, while *L. paracasei* WIS43 significantly attenuated systemic and colonic concentrations of TNF-α, IL-6, and IL-1β in a dextran sulfate sodium-induced murine colitis model [57,58]. *L. paracasei* strains, including KBL382, have additionally been shown to promote IL-10 secretion and drive expansion of the CD4^+^CD25^+^Foxp3^+^ regulatory T-cell compartment within mesenteric lymph nodes, with postbiotic cell-free fractions of the same strain further augmenting mucosal sIgA output—an immunological effector critical for first-line defense against opportunistic oral pathogens [59,60]. Considered in aggregate, the immunomodulatory properties documented across *L. paracasei* strains exhibit a high degree of mechanistic complementarity with the immune–neuroendocrine dysregulation characterizing BMS, which encompasses chronically elevated salivary cortisol and α-amylase, aberrant proinflammatory cytokine profiles, and a permissive mucosal microenvironment that facilitates pathobiont expansion and sustained dysbiosis.

### 4.4. Lacticaseibacillus paracasei: Whole-Genome Insights and Probiotic Profile

The comparative genomics of *L. paracasei* has advanced considerably over the past decade. The foundational pan-genome analysis conducted by Smokvina et al. [23] delineated approximately 4200 orthologous gene clusters across the species, with each genome encoding a conserved core of roughly 1800 genes within a total of 2800–3100 protein-coding sequences. Subsequent whole-genome characterization of strains recovered from ecologically diverse sources—including dairy products, fermented vegetables, palm sap, kefir, and human breast milk—has consistently identified a repertoire of genomic determinants underlying key probiotic-relevant phenotypes: gastrointestinal stress tolerance, mediated by bile salt hydrolase and acid-responsive chaperone systems; mucosal adhesion capacity, conferred by sortase-dependent pilus biosynthesis loci, mucus-binding surface proteins, and exopolysaccharide biosynthetic gene clusters; antimicrobial activity, encoded by sactipeptide and class II bacteriocin operons with specificity against *P. gingivalis* and *S. mutans*; and broad-spectrum carbohydrate catabolic versatility, which collectively reflect the remarkable ecological plasticity of the species across nutritionally heterogeneous environmental niches [23,24,61]. Systematic genomic safety assessment has reproducibly confirmed the absence of horizontally transferable antibiotic resistance determinants and recognized virulence factor genes across examined strains, providing robust genomic corroboration for the GRAS designation assigned to orally administered *L. paracasei* formulations and substantiating their suitability for human clinical application [24]. The probiotic credentials of *L. paracasei* relevant to oral health applications are summarized in Table 3.

### 4.5. Clinical Evidence in BMS and Related Oral Conditions

In interpreting the therapeutic potential of LAB, it is important to distinguish the level of evidence underlying each claim, as the supporting data span a hierarchy of translational maturity. The antimicrobial and immunomodulatory mechanisms described in Section 4.2 and Section 4.3 are derived predominantly from in vitro experiments (e.g., bacteriocin assays, biofilm and postbiotic studies, and cell-culture immunoassays) and, to a lesser extent, from animal models. Evidence of clinical efficacy in humans is currently confined largely to oral conditions adjacent to BMS—such as periodontitis, oral candidiasis, and halitosis—whereas direct interventional evidence in BMS populations themselves remains scarce and preliminary. The distinction between these tiers of evidence—in vitro, animal, related clinical conditions, and BMS-specific clinical data—is made explicit below and should be borne in mind when appraising the translational readiness of LAB-based strategies for BMS. The clinical evidence for LAB-based interventions in BMS and related oral conditions discussed in this section in summarized in Table 4.

Direct evidence from randomized clinical trials evaluating LAB-based interventions specifically in BMS remains limited, though available data are mechanistically plausible and clinically encouraging. In the most methodologically rigorous trial to date, Loncar-Brzak et al. [44] allocated 80 BMS patients across four parallel arms—B-vitamin supplementation, oral probiotic lozenges containing *Limosilactobacillus reuteri* Prodentis, low-level laser therapy, or provision of verbal and written information alone—and documented statistically significant improvements in Oral Health Impact Profile-14 (OHIP-14) quality-of-life scores in both the probiotic and laser therapy arms at one month of follow-up, with no treatment-related adverse events recorded in either group. Complementary and mechanistically supportive evidence is available from a growing body of clinical research examining LAB interventions in conditions of adjacent oral pathology: *L. reuteri* Prodentis lozenges have demonstrated efficacy in reducing clinical indices of gingival inflammation and periodontal pocket depth in patients with chronic periodontitis [45,63,64]; *L. rhamnosus*, *L. acidophilus*, and *Streptococcus salivarius* K12 have each been shown to attenuate oral *Candida* colonization burden in both clinical cohort and experimental model settings [65,66,67]; and multi-strain formulations combining *L. gasseri* with *L. paracasei* have significantly reduced salivary hydrogen sulfide concentrations and total volatile sulfur compound levels in patients with halitosis [68]. Considered collectively, these findings establish a coherent clinical-translational framework that provides both biological rationale and preliminary evidence of efficacy for the design and conduct of BMS-specific, biomarker-anchored randomized trials targeting *L. paracasei* strains as candidate therapeutic agents.

## 5. Mechanistic Links: From Oral Dysbiosis to Neuropathic Pain

### 5.1. Microbe–Immune–Neural Crosstalk in the Oral Mucosa

Communication between the oral microbial community and the trigeminal somatosensory system operates through multiple receptor- and metabolite-dependent pathways. Lipopolysaccharides shed by Gram-negative periodontal pathogens, such as Porphyromonas gingivalis, bind TLR4 on trigeminal nociceptors, stimulating calcitonin gene-related peptide (CGRP) release and NF-κB nuclear translocation; these events heighten neuronal excitability and, over time, may potentially contribute to a chronic pain state [7,69]. Additional pathogenic microbial-derived signals—including N-formyl peptides and pore-forming cytolysins—sensitize TRPV1 and allied ion channels, potentiating nociceptor firing. In contrast, fermentative metabolites, including short-chain fatty acids (SCFAs), indole derivatives, and gamma-aminobutyric acid (GABA), exert opposing anti-nociceptive and anti-inflammatory effects [70].

### 5.2. Short-Chain Fatty Acids, GABA, and the Gut–Brain–Oral Axis

The three principal SCFAs—acetate, propionate, and butyrate—are generated by LAB and allied fermentative bacteria through two anatomically distinct processes: salivary carbohydrate catabolism within the oral biofilm, and dietary fiber fermentation in the colonic lumen. These bioactive molecules transduce their effects through the G protein-coupled free fatty acid receptors FFAR2 (GPR43) and FFAR3 (GPR41), which are expressed on enteroendocrine cells, circulating immune cells, and peripheral sensory neurons. Among the three, acetate and butyrate are capable of traversing the blood–brain barrier via monocarboxylate transporter-mediated uptake, whereupon they modulate central neuroimmune tone by inhibiting histone deacetylase (HDAC) activity and thereby reshaping microglial activation phenotype [70,71]. At the epithelial mucosal surface, SCFAs transcriptionally upregulate structural tight junction proteins—including claudins and occludin—reinforcing paracellular barrier integrity in both the intestinal epithelium and, by anatomical extension, the oral mucosa [62]. Through a mechanistically independent pathway, select *Lactobacillus* strains enzymatically decarboxylate glutamate to produce GABA via glutamate decarboxylase; prolonged colonization with such strains has been shown to remodel GABA receptor subunit expression in cortical and limbic brain regions, yielding quantifiable anxiolytic and antinociceptive behavioral outcomes in rodent models [72,73].

**Figure 5:**
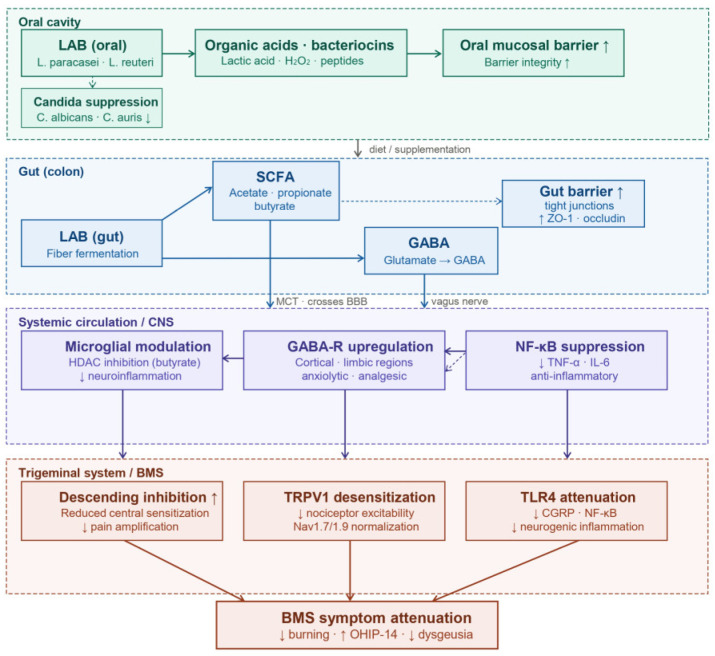
The Oral–Gut–Brain Axis in BMS: A systemic framework for microbial-mediated pain modulation.

Within the pathophysiological context of BMS, these mechanistic threads converge to support a biologically coherent oral–gut–brain axis model: dysbiotic compositional shifts in the BMS salivary microbiome may collectively diminish local SCFA and GABA biosynthetic output, compromise trigeminal mucosal barrier integrity, amplify TLR-mediated peripheral nociceptor sensitization, and ultimately may be associated with the establishment and maintenance of central sensitization—each of these steps representing a discrete and potentially tractable target for LAB-based therapeutic intervention (as synthesized in Figure 5). Extending this framework to central nervous system endpoints, Siviero et al. [74] have additionally implicated olfactory bulb neuroplasticity in BMS pathophysiology, identifying a CNS-level target that may itself be amenable to modulation through systemic and mucosal microbial inputs.

### 5.3. LAB-Derived Metabolites as Anti-Nociceptive Agents

Beyond SCFAs and GABA, a broader repertoire of LAB-derived bioactive compounds—encompassing conjugated linoleic acid (CLA), strain-specific exopolysaccharides, indole-3-aldehyde, phenyllactic acid, and ribosomally synthesized bacteriocins—has been shown to possess antinociceptive, anti-inflammatory, and immune-tolerogenic properties through mechanistically distinct pathways [75,76,77]. Among LAB species, *L. paracasei* strains are particularly notable for their capacity to produce CLA and structurally complex exopolysaccharides at comparatively high yields, both of which have independently demonstrated anti-inflammatory activity in experimental models at the mucosal and systemic levels [54]. The aggregate of these microbially elaborated metabolic outputs constitutes a pharmacologically tractable postbiotic armamentarium—one that, if appropriately formulated, could be deployed therapeutically in BMS with a potentially favorable safety profile, circumventing the theoretical engraftment risks and regulatory complexities inherent to live-organism probiotic administration.

## 6. Future Directions and Knowledge Gaps

### 6.1. Limitations of the Current Evidence

Before outlining future directions, it is essential to critically appraise the limitations of the evidence synthesized in this review, as these constraints temper the strength of the conclusions that can presently be drawn. First, there is substantial heterogeneity in the definition and diagnostic criteria of BMS across studies. Although the ICHD-3 and ICOP frameworks have improved nosological consistency, many of the microbiome and interventional studies cited here predate or did not uniformly apply these criteria, and they vary in their distinction between primary and secondary BMS. This diagnostic heterogeneity limits the comparability of findings across cohorts and may partly account for the inconsistent microbial signatures reported. Second, the oral microbiome studies in BMS are few in number and constrained by small sample sizes, with most enrolling fewer than 50 participants per group; such studies are vulnerable to type II error, inflated effect-size estimates, and limited generalizability. Methodological variability among these studies—including differences in sampling site (saliva, tongue dorsum, mucosal swab), DNA extraction protocols, sequencing platforms, hypervariable region targeted, and bioinformatic pipelines—further complicates cross-study comparison and meta-analytic synthesis. Third, the mechanistic evidence supporting LAB, and *L. paracasei* in particular, is derived predominantly from in vitro experiments and animal models, or extrapolated from other oral and systemic conditions such as periodontitis, oral candidiasis, and inflammatory bowel disease; direct clinical evidence in BMS populations remains scarce. The few interventional studies conducted in BMS are limited methodologically—most notably, the randomized trial by Loncar-Brzak et al. [44] combined probiotics with multiple co-interventions (B vitamins, low-level laser therapy, and verbal/written information) and lacked a true placebo-controlled probiotic arm, precluding attribution of any benefit specifically to the probiotic component. Fourth, the cross-sectional and observational design of nearly all available microbiome studies precludes determination of causal direction; it remains unresolved whether dysbiosis contributes to BMS pathogenesis, arises secondary to altered salivary composition, medication use, or dietary changes in affected patients, or reflects uncontrolled confounding by factors such as age, sex, menopausal status, smoking, and psychiatric comorbidity. Collectively, these limitations indicate that the proposed associations between oral dysbiosis and BMS, and the therapeutic rationale for LAB, should be regarded as hypothesis-generating rather than established, and interpreted with appropriate caution.

### 6.2. Priority Research Directions

Despite encouraging mechanistic and clinical signals, the BMS–microbiome–LAB triangle remains incompletely explored. We identify five priority research directions:

First, adequately powered, randomized, double-blind, placebo-controlled trials of strain-specific LAB interventions—particularly *L. paracasei* strains validated by whole-genome sequencing—are needed in well-characterized BMS cohorts stratified by IASP somatosensory phenotype, psychiatric comorbidity, and microbiome baseline. Endpoint panels should include pain intensity (VAS, BPI), oral health-related quality of life (OHIP-14), salivary flow, biomarker panels (cortisol, α-amylase, cytokines), and 16S/shotgun microbiome characterization at baseline and follow-up.

Second, the current evidence base is predominantly cross-sectional and associative; prospective longitudinal studies and interventional designs are needed to establish the directionality and causal relevance of oral dysbiosis in BMS pathophysiology. Mechanistic studies should clarify the bidirectional relationships between oral dysbiosis and trigeminal small-fiber neuropathy in BMS, leveraging tongue biopsy with intra-epidermal nerve fiber density quantification, corneal confocal microscopy, and matched salivary metabolomics.

Third, the role of *Candida* species—particularly emerging multidrug-resistant species such as *C. auris*—in BMS phenotypes warrants systematic re-investigation using species- and strain-level molecular identification, with parallel evaluation of *L. paracasei* postbiotic preparations as antifungal-sparing interventions.

Fourth, the oral–gut–brain axis remains a hypothesis-rich but evidence-limited domain in BMS. Integrated multi-omics studies (oral and stool microbiome, salivary and serum metabolomics, neuroimaging) are required to move from association to mechanism.

Fifth, postbiotic and engineered-LAB approaches—exploiting defined bacteriocins, exopolysaccharides, and metabolite preparations—may offer pharmacologically tractable alternatives to live-organism administration, with fewer safety concerns and greater regulatory clarity.

## 7. Conclusions

Burning mouth syndrome remains a clinically challenging, mechanistically heterogeneous chronic orofacial pain disorder for which conventional pharmacotherapy delivers only partial relief. Converging associative evidence from salivary microbiome surveys and metabolomic profiling, contextualized within established frameworks of neuropathic pain neurobiology and mucosal immunology, raises the hypothesis that oral microbial dysbiosis constitutes a biologically plausible and potentially modifiable contributor to BMS pathophysiology. It bears emphasis that the existing microbiome data are predominantly cross-sectional and associative in nature; direct evidence for a causal role of oral dysbiosis in BMS etiology remains to be established through longitudinal and interventional study designs. Within this evidence landscape, targeted modulation of the oral microbiome with lactic acid bacteria—particularly *L. paracasei*—may offer a mechanistically coherent and clinically testable therapeutic avenue. Whole-genome sequencing of *L. paracasei* strains, combined with a robust evidence base of antimicrobial, immunomodulatory, and barrier-protective properties, positions *L. paracasei* as a compelling candidate for next-generation BMS clinical trials. Realization of this therapeutic potential will require strain-specific, biomarker-anchored, adequately powered randomized controlled trials conducted within an integrative oral–gut–brain framework—investigations that this review is intended to motivate and inform.

## Figures and Tables

**Table 1:** Summary of major BMS management modalities, their proposed mechanisms, and current evidence.

Therapy	Proposed Mechanism	Evidence Summary	References
Clonazepam (topical/systemic)	GABAA-receptor potentiation; peripheral and central modulation of nociceptive signaling	Topical clonazepam shows the fastest onset; up to 66–75% improvement in observational and randomized data; long-term use is limited by sedation and dependence risk	[39,40]
Alpha-lipoic acid (300–800 mg/day)	Antioxidant; modulates trigeminal small-fiber regeneration	Symptomatic improvement in ~60% of treated patients; effect attenuates after discontinuation; recent RCT meta-analyses report small-to-moderate effect sizes	[41,42]
Capsaicin (topical/rinse)	TRPV1 desensitization in peripheral nociceptors	Effective in short- and long-term studies, particularly in patients with prominent neuropathic features	[7,43]
Cognitive-behavioral therapy	Reduction in pain catastrophizing; modulation of central pain processing	Sustained benefit in patients with comorbid anxiety/depression; low adverse-effect burden	[26,38]
Low-level laser therapy	Photobiomodulation of inflammation, mitochondrial function, and nerve regeneration	Beneficial across multiple RCTs; effect comparable to capsaicin in some comparisons	[38,44]
Probiotics (LAB)	Restoration of microbial balance; antimicrobial, immunomodulatory, and barrier-protective effects	Emerging RCT evidence (e.g., *L. reuteri* Prodentis) shows OHIP-14 quality-of-life improvement; further trials needed	[44,45]

**Table 2:** Summary of oral microbiome studies in burning mouth syndrome.

Sample Size (BMS/Control)	Sample Type	Method	Increased Genera	Decreased Genera	Alpha Diversity	Notes	References
19/22	Unstimulated saliva	16S rRNA V3–V4	*Streptococcus* *Rothia* *Neisseria* *Granulicatella*	*Prevotella* *Haemophilus* *Fusobacterium*	NR	Primary BMS only; first large-scale salivary microbiome study in BMS	[16]
40/40	Saliva	16S rRNA + metabolomics	NR	NR	Reduced (Shannon, Chao1)	BMS with psychiatric symptoms; dysregulated amino acid and nucleotide metabolism	[17]
60/30	Saliva	16S rRNA	*Prevotella* (high-pain) *Alloprevotella* (high-pain)	NR	NR	Pain-stratified; high-pain vs. low-pain BMS vs. healthy controls	[47]
34/30	Saliva	Metabolomics (NMR/MS)	NR	Caffeine metabolism l-DOPA, l-tyrosine, tyramine	NR	Metabolomic profiling; functional correlate of dysbiosis; not a direct microbiome study	[37]

NR = not reported. Alpha diversity reductions are most consistent in subgroups with psychiatric comorbidity or high pain intensity [17].

**Table 3:** Summary of the probiotic credentials of *Lacticaseibacillus paracasei* relevant to oral health applications.

Probiotic Feature	Mechanism/Evidence	References
GRAS/safety	FDA-recognized GRAS status; absence of pathogenicity, transmissible AMR, and biogenic-amine genes in sequenced strains; γ-hemolytic, DNase-negative	[24,51]
Adhesion to oral/gut epithelium	Sortase-dependent pili, mucus-binding proteins, exopolysaccharide biosynthesis clusters; Caco-2 adhesion validated	[23,24]
Anti-*Candida* activity	Inhibition of *C. albicans*, *C. tropicalis*, *C. auris* planktonic cells, biofilms, and persister cells; postbiotic activity retained	[50,51]
Antibacterial activity	Bacteriocin-mediated inhibition of *P. gingivalis*, *S. mutans*; cell-free supernatant suppression of biofilm gene expression	[52,54]
Immunomodulation	TLR2/NF-κB-dependent suppression of TNF-α and IL-6; induction of IL-10 and Foxp3+ Treg populations; IgA stimulation	[56,57]
Mucosal-barrier protection	Upregulation of tight-junction proteins; restoration of intestinal/oral mucosal barrier function in dysbiotic states	[54,62]
Clinical evidence (oral)	RCT evidence for *L. paracasei*-containing complexes in halitosis; supportive evidence in caries prevention and *Candida* control	[46,62]

**Table 4:** Summary of clinical evidence for LAB-based interventions in BMS and related oral conditions.

LAB Strain(s)	Condition	Design	N	Delivery Form	Duration	Primary Outcome	Key Result	Evidence Level	Reference
*Limosilactobacillus reuteri* Prodentis	Burning mouth syndrome (BMS)	RCT, 4-arm parallel	80	Oral lozenges	1 month	OHIP-14 quality-of-life score	Significant improvement in OHIP-14 in the probiotic arm vs. the information-only control; no adverse events reported	RCT	[44]
*L. reuteri* Prodentis	Chronic periodontitis	RCT, crossover	NR	Chewing gum	3 weeks	Gingival crevicular fluid inflammatory mediators	Reduced IL-1β and PGE2 in gingival crevicular fluid	RCT	[63]
*L. reuteri* Prodentis	Chronic periodontitis	RCT, adjunct to SRP	NR	Oral lozenges	3 months	Pocket depth, gingival inflammation	Reduced pocket depth and gingival index vs. SRP alone	RCT	[64]
*L. reuteri* Prodentis	Chronic periodontitis	RCT, double-blind, placebo-controlled	NR	Oral lozenges	12 weeks	Periodontal parameters	Improved periodontal health in military personnel; significant reduction in bleeding on probing	RCT	[45]
*L. rhamnosus*, *L. acidophilus*	Oral candidiasis (elderly)	Clinical intervention	NR	Oral probiotic	NR	Oral *Candida* burden, salivary IgA anti-*Candida*	Reduced *Candida* colonization; increased salivary IgA anti-*Candida*	Clinical study	[65]
Multiple LAB strains (mixed)	Oral candidiasis	Systematic review and meta-analysis	NR	Various	Various	Oral *Candida* burden; prophylaxis/treatment efficacy	Probiotics effective for both prophylaxis and treatment of oral candidiasis; safe profile	Level I (SR/MA)	[66]
Multiple LAB strains (mixed)	Oral candidiasis	Systematic review	NR	Various	Various	Efficacy of probiotics for oral candidiasis management	Probiotics show clinical benefit for oral candidiasis management; evidence supports adjunctive use	Level I (SR)	[67]
*L. gasseri*, *L. paracasei* complex	Halitosis	RCT, double-blind, placebo-controlled	NR	Oral probiotic	NR	Volatile sulfur compounds (VSC): H_2_S, total VSC	Significant reduction in H_2_S and total VSC; novel insights into glucose and phosphorus metabolism	RCT	[68]
*L. paracasei* DSMZ16671	Dental caries prevention	RCT	NR	Oral probiotic	NR	Caries inhibition; safety	Caries inhibition demonstrated; *L. paracasei* confirmed safe for oral use	RCT	[46]

NR = not reported; RCT = randomized controlled trial; SR = systematic review; MA = meta-analysis; SRP = scaling and root planing; OHIP-14 = Oral Health Impact Profile-14; VSC = volatile sulfur compounds. Studies are ordered by condition (BMS, periodontitis, oral candidiasis, halitosis, caries). Evidence level reflects the study design as reported.

## Data Availability

No new data were created or analyzed in this study. Data sharing is not applicable to this article.
